# A Pediatric Case of Hypokalemic Periodic Paralysis With Fatigue and Myalgia

**DOI:** 10.7759/cureus.99022

**Published:** 2025-12-12

**Authors:** Mio Horie, Tatsuo Fuchigami, Tadayasu Kawaguchi, Mika Ishige, Ichiro Morioka

**Affiliations:** 1 Department of Pediatrics, Itabashi Medical System (IMS) Fujimi General Hospital, Fujimi, JPN; 2 Department of Pediatrics and Child Health, Nihon University School of Medicine, Tokyo, JPN

**Keywords:** child, fatigue, hypokalemia, myalgia, periodic paralysis

## Abstract

Periodic paralysis is a rare genetic disorder characterized by recurrent episodes of sudden skeletal muscle weakness. Various factors can trigger this condition, including cold temperatures, abnormal potassium levels, physical activity, and consumption of certain foods. Periodic paralysis is considered benign and usually manifests as muscle weakness. Adult patients with hypokalemic periodic paralysis often experience symptoms such as myalgia and fatigue, whereas myalgia and fatigue are less common in children.

We encountered a pediatric patient with hypokalemic periodic paralysis who presented with weakness, general fatigue, and widespread myalgia.

The patient was a girl ten years and six months old, admitted to our hospital with generalized muscle weakness after exercise. Her maternal relatives exhibited similar symptoms, including weakness. The patient had hypokalemia but normal thyroid hormone levels. After her serum potassium level was corrected with infusion, her trunk strength and her symptoms resolved. Genetic testing revealed a mutation in CACNA1S, leading to a diagnosis of hereditary hypokalemic periodic paralysis. Occasionally, she experienced persistent general fatigue and widespread pain in her neck, shoulders, arms, lower back, and legs without paralysis or hypokalemia for several days.

In conclusion, adult patients with periodic hypokalemic paralysis often experience myalgia and fatigue. Although myalgia and fatigue are rare in pediatric patients, periodic paralysis should still be considered in the differential diagnosis, even in children who show symptoms of muscle pain and fatigue without actual muscle weakness.

## Introduction

Skeletal muscle channelopathies encompass a diverse range of rare muscle disorders characterized by typical symptoms of muscle weakness and myotonia. Myotonia is an impairing disorder characterized by delayed relaxation of skeletal muscles after voluntary contraction, leading to stiffness.

Generally considered benign, these conditions exhibit episodic weakness, with the heart and lungs typically remaining unaffected [1]. They are classified into two categories based on notable clinical symptoms: nondystrophic myotonia and periodic paralysis (PP). PP is a genetic disorder known to cause recurrent episodes of paroxysmal skeletal muscle weakness and paralysis triggered by various factors such as cold temperatures, fluctuating potassium levels, exercise, and certain foods. This arises from mutations in various ion channel genes in skeletal muscle. SCN4A and CACNA1S genes encode the alpha subunits of the voltage-gated sodium and calcium channels, respectively, which are abundant in muscles used for movement. These channels are crucial for the proper function of skeletal muscles, and mutations in either gene can cause disorders like PP, characterized by episodes of muscle weakness. PP consists of two types, defined by serum potassium levels during paralytic attacks: hyperkalemic periodic paralysis (HyperPP) and hypokalemic periodic paralysis (HypoPP). Mutations in the sodium channel (SCN4A) gene are responsible for HyperPP, whereas mutations in two other genes lead to HypoPP, specifically SCN4A (HypoPP2) or the skeletal muscle calcium channel (CACNA1S) gene (HypoPP1) [2].

PP is considered limited to episodes of muscle weakness [3]. However, adult patients with HypoPP often experience symptoms such as myalgia (muscle pain) and fatigue, and fibromyalgia-like pain has also been reported in patients with PP [3-4]. There have been a few reports of adult PP patients with these symptoms, but pediatric patients showing myalgia and fatigue are rare [5, 6]. We treated a pediatric patient with HypoPP who exhibited not only weakness but also general fatigue and widespread muscle pain.

## Case presentation

The patient was a 10-year-and-6-month-old Japanese girl. She was a fifth-grade elementary school student who played tennis once a week. She was admitted to our hospital with generalized weakness. Three days prior to admission, she had run approximately 13 laps around the schoolyard in practice. Two days prior to admission, she had played badminton and soccer for approximately 30 min. The day before admission, she played badminton and performed horizontal bar exercises for about one hour. In the evening, the patient developed upper limb pain and mild weakness in her lower limbs. After she went to bed, the weakness spread throughout her body. On the day of admission, she visited her local doctor, who referred her to our department for treatment.

Her medical history showed that a year previously, she had experienced walking disturbance due to low extremity muscle weakness and was referred to her local doctor for further examination and treatment at Nihon University Hospital. At the time of examination, her symptoms had mostly subsided, and blood tests revealed no abnormalities in electrolytes, liver enzymes, or thyroid hormones. Urinalysis showed no electrolyte issues, and CSF examination for excluded Guillain-Barré syndrome revealed no elevated cell counts or protein cell dissociation. Contrast-enhanced MRI of the spinal cord revealed no abscesses or macular lesions. However, QT prolongation was observed on electrocardiography (ECG), and the underlying cause remained unknown. The patient was discharged on the seventh day after spontaneous recovery.

**Figure 1:**
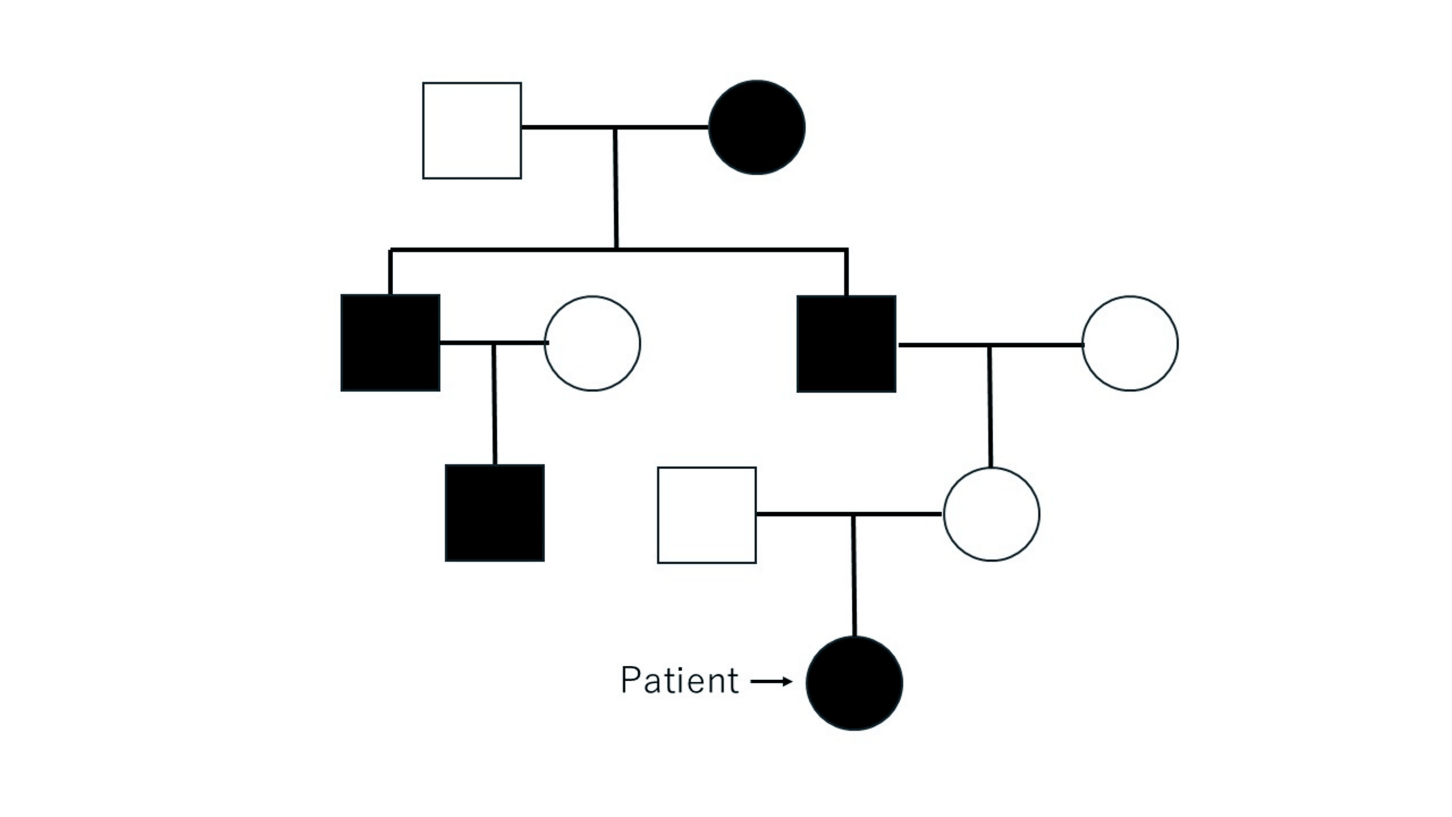
Family Pedigree Family pedigree with males and females represented by squares and circles, respectively. Affected and unaffected individuals are represented by filled and open symbols, respectively. The arrow indicates the patient.

In her family history (Figure 1), her maternal grandfather, her grandfather’s brother and his son, as well as her great-grandmother, exhibited similar symptoms, including generalized weakness.

Due to her quick recovery from symptoms and her family history, the suspected diagnosis was PP, and she was monitored with an ECG for QT prolongation three to four times a year at the outpatient clinic. However, her ECG results showed no QT prolongation.

At the time of her admission to our hospital, the patient was 138.5 cm tall (-0.3 SD) and weighed 30.1 kg (-0.6 SD). The body mass index (BMI) was 15.7. Her body temperature measured 36.7°C, her heart rate was 56 beats per minute, and her blood pressure was 123/94 mmHg. She exhibited generalized weakness, was unable to stand, and remained seated in a wheelchair due to truncal weakness while leaning against it. Her consciousness was clear, and her speech was coherent. She could take fluids, and she showed no signs of respiratory distress or dysphagia. Her facial color was normal. Chest and abdominal examination revealed no abnormalities. A neurological assessment revealed reduced patellar tendon reflexes. A manual muscle strength test showed grade 1 strength (only muscle contraction, no joint movement) in the neck, trunk, deltoid, biceps, triceps, and lower-extremity muscles. Although her grip strength was not tested, she was able to perform a release handshake.

Table 1 shows the laboratory findings at admission. Blood tests revealed low serum potassium levels (2.3 mEq/L) and high serum creatinine kinase levels (337 U/L).

**Table 1:** Laboratory findings on admission ALP: alkaline phosphatase; ALT: alanine aminotransferase; AST: aspartate aminotransferase; APTT: activated partial thromboplastin time; BG: blood glucose; BUN: blood urea nitrogen; Ca: calcium; CK: creatinine kinase; CL: chlorine; CRP: C-reactive protein; ESR: erythrocyte sedimentation rate; F-T3: free triiodothyronine; F-T4: free thyroxine; HCO3: hydrogen carbonate; HDLC: high-density lipoprotein cholesterol; IP: inorganic phosphorus; K: potassium; LDH: lactate dehydrogenase; LDLC: low-density lipoprotein cholesterol; Na: sodium; PH: potential hydrogen; PT: prothrombin time; RBC: red blood cell; TC: total cholesterol; WBC: white blood cell

Hematologic Test				Biochemical Test				Urinary Test			
Test	Result	Reference Range	Units	Test	Result	Reference Range	Units	Test	Result	Reference Range	Units
WBC	119	(33–86)	10^2^/μL	CRP	0	(0.01–0.14)	mg/dL	PH	5.5	(5.0–8.0)	
Neutrophil	85.6		%	Total Protein	6.9	(6.6–8.1)	g/dL	Specific gravity	1.021	(1.002–1.030)	
Eosinophil	0.2		%	Albumin	4.6	(4.1–5.1)	g/dL	Protein	(-)		
Basophil	0.8		%	CK	337	(40–165)	U/L	Glucose	(-)		
Monocytes	3.3		%	AST	28	(8–40)	U/L	Ketone	(-)		
Lymphocytes	10.1		%	ALT	16	(5–45)	U/L	Na	227		mEq/L
RBC	496	(386–492)	10^4^/μL	LDH(IFCC)	275	(124–222)	U/L	K	17		mEq/L
								CL	311		mEq/L
								Creatinine	70.82		mg/dL
Hemoglobin	13.6	(11.6–14.8)	g/dL	ALP	288	(38–113)	U/L				
Hematocrit	42.7	(35.1–44.4)	%	BUN	8.5	(8.0–23.0)	mg/dL				
Platelet	25.9	(15.8–34.8)	10^4^/μL	Creatinine	0.33	(0.46–0.80)	mg/dL				
Coagulation test				Na	141	(136–147)	mEq/L				
PT sec	11.7	(10.5–13.0)	sec	K	2.3	(3.5–5.0)	mEq/L				
PT %	91.8	(80–125)	%	CL	108	(98–108)	mEq/L				
PT ratio	1.07			Ca	9.6	(8.5–10.2)	mg/dL				
PT INR	1.07	(0.75–1.15)		IP	2.6	(2.5–4.5)	mg/dL				
APTT sec	25.6	(20–40)	sec	BG	113	(70–109)	mg/dL				
ESR	8	(0–15)	mm/h	TSH	0.61	(0.34–3.88)	μIU/ml				
Fibrinogen	204.8	(200–400)	mg/dL	F-T3	4.65	(2.13–4.07)	pg/dL				
D-Dimer	0.5	(0.0–1.0)	μg/mL	F-T4	1.22	(0.95–1.74)	ng/dL				

Thyroid hormone levels were within normal ranges. The fraction of excreted potassium (FeK) was 3.5%, which is < 10%, indicating no excessive urinary potassium excretion [7]. ECG showed a QTc of 0.46, compared with the reference values of 0.430 for a first-grade elementary school girl and 0.455 for a first-year junior high school girl, using Fridericia’s formula, suggesting QT prolongation [8].

After admission, the patient was connected to an ECG monitor, and her serum potassium level was corrected with an infusion for follow-up. Nineteen hours after admission, her serum potassium levels normalized, her trunk strength improved, and her symptoms showed signs of improvement. On the second day of hospitalization, the QT prolongation improved, and no QT prolongation was observed during treadmill exercise. Her symptoms continued to improve on the third day, and the infusion was discontinued. She was discharged on the fourth day.

At the post-discharge stage, a genetic test conducted at Nihon University Hospital revealed an etiopathogenic heterozygous c.1583G>A (p.Arg528His) mutation in the calcium channel gene CACNA1S by direct Sanger sequencing, which led to a diagnosis of hereditary HypoPP.

We advised the patient to be cautious during general anesthesia and to understand her limitations while monitoring her condition, as excessive exercise and a diet high in carbohydrates are thought to have triggered the disease. She was followed up as an outpatient.

**Figure 2:**
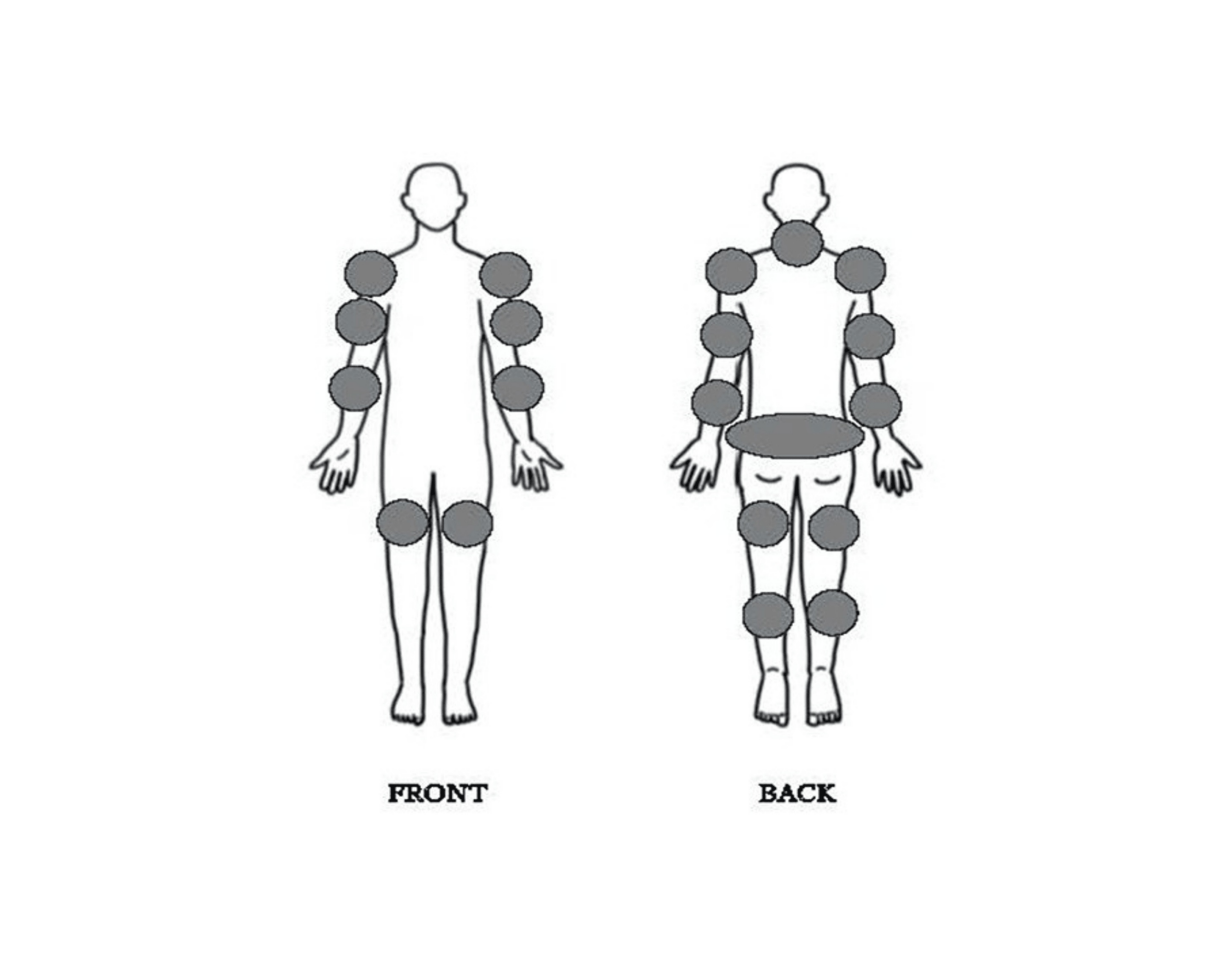
Body pain map for the patient Dark colors represent regions of pain.

After discharge from the hospital, the attacks became less severe than those at admission; however, mild weakness was noted once every 1-3 months, usually after activities like schoolyard running, rope-jumping competitions, and trips. Her serum potassium level stayed within the normal range, no ECG abnormalities were observed, and her symptoms improved spontaneously within a few hours; therefore, she was not readmitted. Nevertheless, she occasionally experienced persistent general fatigue and widespread pain for several days without paralysis, hypokalemia, or elevated serum CK levels. After 10 minutes walking, her myalgia, notably lower leg muscles, worsened, and she was admitted to the hospital for a rest for 3 days. Her pain was mainly felt in the neck, upper arms, shoulders, lower back, and lower extremities (Figure 2).

Owing to the increasing frequency of symptoms related to body pain, we started oral acetazolamide (4-12.5 mg/kg/day) as a preventive medication. Although the frequency and intensity of her paralytic attacks improved with acetazolamide treatment, her body pain did not improve, and her medication was changed to potassium aspartate (300 mg), six tablets per day. After the medication was changed, her general fatigue and body pain showed mild improvement. Nonetheless, she occasionally experienced muscle weakness, overall fatigue, and widespread body pain after engaging in activities such as school physical exercise, forgetting to take medication, or overeating.

## Discussion

Primary PP includes HypoPP, HyperPP, and Andersen-Tawil syndrome. The estimated prevalence of these conditions is approximately 1 in 200,000 for HyperPP, 1 in 100,000 for HypoPP, and 1 in 1,000,000 for Andersen-Tawil syndrome, meaning HypoPP is the most common type of PP [9-11]. Sasaki et al. reported a spectrum of mutations in Japanese patients with HypoPP1 and HypoPP2. However, the frequencies of HypoPP1 (57%) and HypoPP2 (43%) in Japan differ from those reported in European countries, which range from 70% to 90% and 10% to 25%, respectively [2].

Diagnostic criteria (Table 2) proposed by Sansone et al. are used to diagnose HypoPP. These requirements do not include the symptoms of myalgia and fatigue. Although there have been a few reports of adult PP patients with these symptoms, pediatric patients exhibiting myalgia and fatigue are rare [3]. Neame et al. reported the case of a nine-year-old boy who was diagnosed with HypoPP after experiencing persistent fatigue and muscle aches associated with mildly elevated creatine kinase (CK) levels [5].

**Table 2:** Consensus diagnostic criteria of hypokalemic periodic paralysis [13]

1.	Two or more attacks of muscle weakness with documented serum potassium<3.5 mEq/L
	OR
2.	One attack of muscle weakness in the proband and one attack of weakness in one relative with documented serum potassium <3.5 mEq/L
	OR
3.	Three or more of the following six clinical and laboratory features:
a.	Onset in the first or second decade
b.	Duration of attack (muscle weakness involving > 1 limb) longer than two hours
c.	Presence of triggers (previous carbohydrate-rich meals, symptom onset during rest after exercise, and stress)
d.	Improvement in symptoms with potassium intake
e.	Family history of the condition or genetically confirmed skeletal calcium or sodium channel mutation
f.	Positive McManis long exercise test [12]
	And
4.	Exclusion of other causes of hypokalemia (renal, adrenal, thyroid dysfunction, renal tubular acidosis, diuretic, and laxative abuse)

Fibromyalgia is characterized by widespread musculoskeletal pain, fatigue, sleep problems, and memory and mood issues. Götze et al. reported that a mother and son with HyperPP experienced fibromyalgia-like pain [4]. In a study by Giacobbe et al., 44 adult patients (71% female, with an average age of 47.6 years) with PP, including HypoPP (84%), HyperPP (14%), and others, reported body pain as a common symptom. This pain is most noticeable in the neck, lower back, and lower extremities, and 55.9% of patients with HypoPP meet the criteria for fibromyalgia. The PP patients who met the criteria for fibromyalgia or moderate/severe pain also had coexisting fatigue, depression, and poor sleep quality. The rate of depression and poor sleep in PP patients was higher than in the general population [3]. In another study, adult patients aged over 40 years with PP (average age, 60 years) reported pain in 86% of HypoPP and 67% of HyperPP cases [14]. The mechanism by which pain occurs in PP remains unclear. However, the overlap case between a predominantly myalgic phenotype and the PP phenotype, and the underlying myopathy in cases of elevated CK were suggested [3,5].

The pediatric patient in this study experienced widespread pain and fatigue without paralysis. Her body pain map showed areas in the neck, lower back, and lower extremities that closely resembled those reported by Giacobbe et al. [3].

Carbonic anhydrase inhibitors (especially acetazolamide and dichlorphenamide) have been used empirically for both hypoPP and hyperPP [15]. Our preventive pharmacological therapy included carbonic anhydrase inhibitors, particularly acetazolamide, and oral potassium. The acetazolamide treatment effectively reduced the frequency and severity of her paralytic attacks, but her body pain did not improve. For bodily pain, potassium aspartate medication, used to maintain serum potassium levels, was more effective than acetazolamide.

Adults with various neuromuscular diseases have validated their quality of life using the Individualized Neuromuscular Quality of Life (INQoL) scale [16]. The comparison of INQoL subscales in PP patients between Sasaki’s study in Japan and the latest study in the U.S. showed that domains related to “symptoms”, including pain, fatigue, and myotonia, were milder in Japan than in the USA [2,17,18]. The patient's pain and fatigue were mild in this study. Previous reports have indicated that myalgia and fatigue are common in patients with PP. Myalgia and fatigue significantly impact quality of life. Therefore, myalgia and fatigue are considered key symptoms of PP.

A limitation of this study is that it is a single case report; more case studies are needed.

## Conclusions

We examined a pediatric case of HypoPP presenting with myalgia and fatigue.

Adult patients with HypoPP often exhibit symptoms such as myalgia and fatigue. PP should be considered in the differential diagnosis of patients presenting with these symptoms, especially if there is a family history or triggers like exercise or a carbohydrate-rich diet. Although myalgia and fatigue are rare in pediatric patients, PP should still be included in the differential diagnosis, even in children who present with muscle pain and fatigue without actual muscle weakness.
